# A review of the actions of Nitric Oxide in development and neuronal function in major invertebrate model systems

**DOI:** 10.3934/Neuroscience.2019.3.146

**Published:** 2019-08-19

**Authors:** Nicholas J. D. Wright

**Affiliations:** Associate professor of pharmacy, Wingate University School of Pharmacy, Wingate, NC28174, USA

**Keywords:** nitric oxide, neuronal development, neuronal function, insect, mollusc, crustacean

## Abstract

Ever since the late-eighties when endothelium-derived relaxing factor was found to be the gas nitric oxide, endogenous nitric oxide production has been observed in virtually all animal groups tested and additionally in plants, diatoms, slime molds and bacteria. The fact that this new messenger was actually a gas and therefore didn't obey the established rules of neurotransmission made it even more intriguing. In just 30 years there is now too much information for useful comprehensive reviews even if limited to animals alone. Therefore this review attempts to survey the actions of nitric oxide on development and neuronal function in selected major invertebrate models only so allowing some detailed discussion but still covering most of the primary references. Invertebrate model systems have some very useful advantages over more expensive and demanding animal models such as large, easily identifiable neurons and simple circuits in tissues that are typically far easier to keep viable. A table summarizing this information along with the major relevant references has been included for convenience.

## 1. Introduction

Ever since the late-eighties when endothelium-derived relaxing factor (EDRF) was found to be the gas nitric oxide (NO) [1]–[3] endogenous NO production has been observed in virtually all animal groups tested and additionally in plants, diatoms, slime molds and bacteria [4]–[8]. The fact that this new messenger was actually a gas and therefore didn't obey the established rules of neurotransmission made it even more intriguing. Research into this novel new player has expanded rapidly especially in the field of neuroscience with NO being implicated in such significant and wide-ranging processes as olfaction, learning and memory and dementia. In neuroscience invertebrate models have always been held in high regard because the nervous systems are simpler and typically composed of large peripherally-arranged cell bodies that can often be identified from preparation to preparation so ensuring the utilization of the same neuron in each experiment. This review is focused on the direct effects of NO on invertebrate neurons and nervous systems and additionally neuronal developmental in which NO has been implicated. A review of all the invertebrate organisms utilized for NO research, let alone all animals, would probably be overwhelming so for this review the groups covered will be limited to the major model organisms from the molluscs, insects and their watery cousins the crustaceans. This still leaves a significant number of organisms to cover especially as, for example, there are several different species of land snail utilized by researchers around the world. The author himself used *Helix aspersa* because they were easy to collect locally (and free!) in and around the University of Southampton in England where he performed his PhD. under the direction of Dr. R.J. Walker. Where possible the individual species are discussed but if too numerous they are grouped appropriately. Hopefully the majority of research utilizing these animals has been included and additionally compiled into a comprehensive table for easy reference (Table 1). It is hoped that this review can help guide the reader to the primary literature on the appropriate model system for a detailed description which would be beyond the scope of this review.

## 2. Nitric oxide

Before discussing the possible effects of NO on these selected invertebrates' neurons and nervous systems, it might prove useful to briefly discuss the origins and chemistry of NO; for a far more comprehensive overview the reader is recommended to access Moroz and Kohn's excellent 2011 review [9]. That review suggests NO involvement in signaling is traceable back to the origins of life. Nitric oxide is infact part of the nitrogen cycle and a vital intermediate which is far more reactive than nitrogen itself which has to be “fixed” before most organisms can utilize it [10]–[15]. It should be noted that “nitric oxide” actually includes the nitrosyl radical itself plus the nitroxyl and nitrosonium ions [16]–[19]. Nitric oxide is quite reactive and can form covalent bonds with many biological molecules including its primary target guanylate cyclase; this is significant in itself as most signal transduction interactions do not involve formal covalent bond formation. As a hydrophobic gas NO can cross biological membranes easily and because of this it is considered to act as a “3-D” volume messenger unlike conventional transmitters that are typically limited to synaptic locations for transmission [20]–[24]. Additionally it can be appreciated that NO levels are affected by the redox status of a cell and there appears to be a complex relationship with oxygen gradients and so-called “metabolic budgets” [25],[26]; this may have been an early function of NO in biological systems. There is data linking NO to mitochondrial function [27]–[36] and its half-life can be quite variable ranging from a few milliseconds to days depending on the chemical environment [9]. Despite this NO is considered a relatively short-range messenger acting in an autocrine and/or paracrine manner. These factors help explain why direct measurement of NO in living tissues is difficult. This has resulted in the development of a plethora of methods for NO detection and quantification. Although not the primary focus of this review it is probably useful to mention and provide references here to the main techniques for NO detection in living tissues. One of the first methods available to invertebrate neurobiologists utilized NO-sensitive electrodes and was rapidly followed by fluorometric detection, spin-trapping and even capillary electrophoresis at the single cell level [37]–[55]. Probably in all invertebrate species examined the first task was not the detection of endogenous NO production but the far easier demonstration of the presumed primary source of NO in biological systems, namely the enzyme nitric oxide synthase (NOS). Initially this was demonstrated by NADPH-diaphorase histochemistry and is invariably cited in virtually all of the physiological function papers tabulated here (Table 1), many of which demonstrated NOS expression almost as a prerequisite to observing any effects NO might have. Again, the reader is referred to other sources for a proper discussion of this technique [56]–[59]. In 1990 Snyder's research group isolated and purified so-called neuronal NOS (nNOS) from rat cerebella and used this to develop antibodies for immunohistochemical localization [60],[61]. Finally it should be noted that there are in fact at least 7 potential enzymatic sources of NO production in living organisms. These include the so-called “classic” multi-domain NOS's found in animals and slime molds [62],[63], a prokaryotic “truncated” NOS found in many bacteria [7],[64] and various nitrite reduction systems [7],[28],[65]–[73].

## 3. Nitric oxide synthase

Despite multiple potential sources, the principal source producing NO in animals, including the subjects of this review, is the family of “classic” multi-domain NOS's. As mentioned, the first constitutive NOS isoform was cloned from rat brain (nNOS) 4 years after the discovery that EDRF was NO in 1991 [74]. One year later 2 other isoforms were cloned; an inducible type (iNOS) from macrophages [75]–[77] and a second constitutive type (eNOS) from endothelium [78]–[82]. All 3 NOS's were similar to cytochrome P450 with reductase and oxygenase domains. It is thought parallel evolution occurred from a single truncated NOS a billion years ago and while iNOS is calcium independent, nNOS and eNOS are calcium-calmodulin dependent [83],[84]. In all animal tissues NOS catalyzes NO production from a reaction between L-arginine and molecular oxygen with the release of L-citrulline [62],[63]. In 1991 the first evidence for the function of NO in invertebrates (Limulus; the horseshoe crab) was published [85] quickly followed the next year by NOS histochemistry revealing widespread expression in several major invertebrate phyla including molluscs and arthropods. Currently it is thought that insects have one NOS gene while molluscs have two [56]. The two calcium-calmodulin dependent NOS's (nNOS and eNOS) are activated by elevated intracellular calcium typically either from ligand- or voltage-gated calcium channels or internal stores. Inducible NOS is expressed and activated in the presence of bacterial lipopolysaccharides or damaging stimuli [9].

**Table 1.** this table attempts to summarize the material discussed in this review including the animals comprising each of the groups, the main research topics studied and the principal references.

	Number	Group	Source Refs	Subjects Covered
**INSECT DEVELOPMENT**				
Locusta migratoria, Schistocerca gregaria (locust)	11	GP1	100–110	Embryonic development, neuronal migration, growth cone function and synaptogenesis.
Drosophila melanogaster (fruit fly)	14	GP2	111–124	Adult development, visual system development, growth cone/filopodial function and tracheal development/response to hypoxia.
Manduca sexta (moth)	8	GP3	125–132	Development at all stages, sensory system development (olfactory and visual), neuronal migration, differentiation and arborization.
Gryllus bimaculatus	1	GP4	133	NO involved in environmentally-induced neurogenesis in the mushroom bodies.
**INSECT NITRIC OXIDE**				
Apis mellifera (honeybee)	5	GP5	134–139	Established learning and memory system/proboscic extension for sucrose reward, NO involved at several levels of olfaction (Mushroom bodies and Antennal lobes).
Locusta migratoria & gregaria (locust)	16	GP6	140–155	NO affects several sensory modalities and motor pattern responses, heart regulation; neuropil architecture suits 3-D “volume transmitter” (= gas).
Manduca sexta, Bombyx mori (moth)	9	GP7	156–164	NO involved in odor perception/short-term memory formation (enhance inward currents), variations with circadian rhythm, interactions with nicotinic receptors.
Drosophila melanogaster (fruit fly)	4	GP8	165–168	Retrograde transmitter at larval neuromuscular junction/vesicle release, NO-cGMP implicated in Antennal lobe function/projection neurons.
Lampyridae (firefly), Neobellieria bullata (fleshfly), Phormia regina (blowfly).	4	GP9	169–172	NO in fireflies controls flashing, cGMP in taste in blowflies and NO in olfaction in fleshflies.
Chorthippus biguttulus (grasshopper)	4	GP10	173–176	No and cGMP involvement in central complex sound production and in juvenile hormone release (reproductive function).
Gryllus bimaculatus (cricket)	6	GP11	178–183	NO implicated in long-term memory formation via cGMP and PKA, affects Mushroom body neurogenesis and Kenyon cell function and may be involved in submissive behavior.
Periplaneta Americana, Blaberus craniifer (cockroach)	6	GP12	148,184–188	Allosterism of sGC, estradiol affects NO production, NO affects nicotinic currents and long-term memory.
**MOLLUSC DEVELOPMENT**				
Lymnaea stagnalis (pond snail)	3	GP13	189–191	NO involved in embryonic development, neurite growth and synaptic re-modelling after injury, NO implicated in locomotion, heartbeat and feeding.
Helisoma trivolvis (pond snail)	11	GP14	122,192–201	NO affects growth cone function via sGC, cGMP, Ca^2+^ (internal source) and PKG; NO chemotactic for pathfinding, affects K^+^ currents and ciliary function plus causes ADP-ribosylation.
Ilyanassa obsoleta (sea snail)	2	GP15	202–203	
**MOLLUSC NITRIC OXIDE**				
Land snails	22	GP16	204–227	Peptides, membrane currents, analgesia, hypoxia, cold, nociception, synaptic and retrograde transmission, olfaction, activity vs rest and memory.
Limax Maximus, Limax Valentianus, Limax Marginatus (land slug)	8	GP17	228–235	NO-cGMP affects olfaction, discrimination and learning and memory in the oscillating procerebrum of this established odor processing model.
Lymnaea stagnalis, Helisoma trivolvis, Planorbarius corneus (pond snails)	25	GP18	215,226,236–258	Feeding behavior and rythmic activity in buccal ganglion, internal Ca^2+^ release, synaptic transmission, long-term memory and conditioning, response to glutamate and iNOS expression.
Stramonita haemastoma (sea snail)	2	GP19	259 & 260	NO associated with sensory afferents and response to environmental stress.
Crenomytilus grayanus, Mytilus edulis, Pecten irradians (bivalve molluscs)	6	GP20	261–266	Transcutaneous electrical nerve stimulation (TENS) system model for pain, neuroprotective mechanisms for temperature, hypoxia and pollution, NO involved in ciliary activity regulation.
**Aplysia (and other sea slugs)**				
Aplysia, Pleurobranchaea californica, Onchidium (sea slugs)	24	GP21	267–289	Feeding/swallowing, NO affects buccal ganglion, modulates/affects dopamine, acetylcholine, glutamate, histamine and Met-encephalin-induced membrane currents plus directly depolarizes and neuropathic pain model.
**Crustacean Development**				
Homarus americanus (lobsters).	2	GP22	290 & 291	NO in development and injury of olfactory system, Stomatogastric ganglion responsiveness, possible involvement in transcription.
**Crustacean Nitric Oxide**				
Homarus americanus (lobster)	4	GP23	292–295	NO and neuropeptides in heart control.
various Crabs	6	GP24	296–301	Pigment in the retina, nociceptive stimulii processing and somatogastric ganglion activity.
Pacifastacus leniusculus (crayfish)	12	GP25	302–313	Glial cell apoptosis (Photodynamic therapy), sensory processing and plasticity and retrograde synaptic transmission at the neuromuscular junction.
Calanus finmarchicus (zooplankton)	1	GP26	314	N/A

## 4. Nitric oxide effectors

As mentioned earlier, the primary mechanism of action of NO is thought to be via the activation of soluble guanylate cyclase (sGC) which produces the secondary messenger cyclic guanosine monophosphate (cGMP) [86]. The term soluble is probably not ideal as at least one isoform of GC is thought to actually bind to PDZ domains of synaptic scaffolding proteins so is not “free” in the cytoplasm as the name implies [87]. Guanylate cyclase is a dimeric enzyme with 2 subunits and an NO-binding heme group [88]. The covalent binding of NO to sGC causes the production of cGMP from GTP [89]. Cyclic GMP can then activate several major types of effectors including cGMP-dependent protein kinases (PKGs), cGMP-gated ion channels and various phosphodiesterases which also degrade cGMP back to GMP; the reader is referred to the review by Francis et al. for an in-depth discussion of this topic [90]. Additionally NO can covalently react with redox-sensitive cysteine residues in many proteins causing S-nitrosylation which can cause, for example, changes in enzyme activity, protein-protein binding, membrane targeting, transport systems and protein folding and stability. Currently around 3000 different affected proteins have been identified [91]–[99].

## 5. Actions of nitric oxide on invertebrate model systems

The remainder of this review will attempt to summarize the actions of NO on selected invertebrate model systems, specifically molluscs, insects and their watery cousins the crustaceans. Additionally the table (Table 1) will present much of this data in a more accessible form complete with selected references. Each “family” (molluscs vs. insects vs. crustaceans) is divided into developmental or direct effects of NO on neurons and nervous systems and then further divided into groups (together with their selected references; see Table 1) of either individual species or closely related species depending on the amount of research performed utilizing that species; there are 26 groups in total.

## 6. Insect development (Groups 1–4)

Group 1 involves developmental research utilizing locusts and the effects of NO (Locusta migratoria and Schistocerca gregaria). In the embryonic locust the NO-cGMP system is permissive for neuronal development and migration (antagonized by carbon monoxide); research using the enteric nervous system has produced videos of actual neuronal migration. It is implicated in the development of the central complex which is thought to be involved in spatial orientation/awareness. Nitric oxide also affects axon growth and regeneration in locusts including growth cone function, synaptogenesis and neuronal maturation [100]–[110].

Group 2 includes development observed in Drosophila melanogaster (fruit fly). The NO-cGMP system is essential for proper development, specifically affecting neuronal proliferation, re-modelling, specificity and differentiation. Nitric oxide-cGMP is strongly implicated in the development of the visual system including effects on growth cone filopodia and may act as a retrograde transmitter at neuromuscular junctions (NMJ's) and in eye development. The NO-cGMP-PKG system is used by fruit flies in response to hypoxia [111]–[124]. Group 3 concerns the moth Manduca sexta. The NO-sGC-cGMP system is implicated in all stages of development from embryo to larva to pupal/adult. Nitric oxide is thought also to be involved in neuronal migration, differentiation and arborization. In particular NO appears necessary for the development of various sensory systems including the visual system and the antennal lobes (AL's)/olfactory system. Nitric oxide also stimulated motorneurons and was involved in the development and migration of the peripheral nerve plexus which gives rise to neurons in the ventral nerve cord in the larval stage. It is worth noting that Gibson et al. reported disruption of AL development after blocking NO-mediated ADP-ribosylation [125]–[132]. Group 4 used the cricket Gryllus bimaculatus as a model system. It may have been more appropriate to place this in group 1 with its larger relatives but Cayre et al. nicely demonstrate that endogenous NO has a key role in environmentally-induced neurogenesis of the mushroom bodies (MB's), structures thought to be involved in associative learning in insects [133].

## 7. Insect physiology (Groups 5–12)

The next 8 groups (5–12) summarize the direct effects of NO on neurons and nervous systems of insects. Group 5 involves research on Apis mellifera (the honey bee). The honey bee is an established associative learning and habituation model system, typically monitoring proboscis extension to a sucrose reward. This response may actually involve activation of PKA as opposed to PKG by the NO-cGMP system. In the AL's NO manipulation can affect odor discrimination. Mushroom body neurons in vitro show increases in intracellular calcium with nitric oxide confirming NO's significance throughout this insect's olfactory system. Nitric oxide also appears crucial for the conversion of short into long term memory [134]–[139]. Group 6 summarizes direct effects of NO on neurons and nervous systems of locusts (Locusta migratoria and Schistocerca gregaria). Here NO has been implicated in several important functions such as affecting motor patterns for feeding and egg laying and regulating the heart. Nitric oxide appears to be involved in several sensory modalities including taste, olfaction and vision; NO is implicated in significant sensory and motor function in the locust. Apparently the chemosensory response to NaCl is regulated by NO as is the response of leg hairs to mechanical stimulation. The response to salt is thought to be cGMP-independent and research involving the cockroach and locust has suggested allosterism occurring in the response of sGC to NO. Interestingly, NO has been shown to affect spreading depression in the locust metathoracic ganglion via the cGMP-PKG system; if activated, spreading depression is increased. Of particular significance is the research that suggests NO's ability to act as a 3-D “volume transmitter,” due to it being a gas, suits the physical arrangement of ganglia neuropil in the locust [140]–[155]. Group 7 utilizes moths to study the effects of NO (2 species here; Manduca sexta and Bombyx mori). This group focuses on olfaction primarily and increases in NO with odor stimulation can be observed in the AL's projection neurons in an apparently sGC-independent manner (an NO-insensitive sGC has been isolated from Manduca). The stimulation causes an increase in inward depolarizing membrane currents. Nitric oxide appears necessary for olfactory short term memory but not for discrimination. Additionally basal NO levels appear to vary with circadian rhythm and that there appears to be a relationship between nicotinic acetylcholine receptors and cGMP levels via NO, probably due to gating voltage-sensitive calcium channels [156]–[164]. Group 8 uses Drosophila melanogaster, typically the larval stage, for NO studies. Detailed characterization of the Drosophila NOS has shown it to be Ca^2+^-calmodulin sensitive and very similar to mammalian nNOS. As per Manduca, a virtually insensitive to NO sGC has also been isolated and may be necessary for a response to hypoxia. Using the established larval wall NMJ preparation, NO has been implicated as a retrograde transmitter capable of increasing cGMP levels in pre-synaptic terminals and enhancing vesicle release in a calcium-independent manner. Nitric oxide is again implicated in AL projection neuron function with increasing NO levels decreasing cholinergic spontaneous excitatory post-synaptic potentials [165]–[168]. The next group (9) contains some rarer but novel fly models (Photinus and Photuris fireflies, the blowfly Phormia regina and the fleshfly Neobellieria bullata). Nitric oxide appears to mediate neuronal control of flashing in fireflies while in blowflies taste receptors may use cGMP for signal transduction. The fleshfly also probably uses NO in olfaction [169]–[172]. Group 10 looks at NO in Chorthippus biguttulus (grasshopper); I have separated locusts, grasshoppers and crickets for the sake of this review. This model was used extensively by the Heinrich group to look at central complex function specifically with respect to producing the appropriate sound production for reproduction. Disruption of the NO-cGMP system affects this function; if the environment is unsuitable for reproduction, elevated NO raises the behavioral threshold for sound production. The endocrine gland the corpora allata releases juvenile hormone also necessary for reproduction; NO and cGMP may be involved with NO possibly acting as a retrograde transmitter [173]–[176]. Group 11 looks at NO in the cricket Gryllus bimaculatus. It was found that long term memory formation may be due to PKA being activated by cGMP via adenylate cyclase and cyclic adenosine monophosphate unlike the situation observed in the honeybee [139],[177]. Mushroom body neurogenesis appears affected by NO which also increases the probability of calcium channel opening in the principal MB neurons the Kenyon cells; the mechanism here may involve PKG. Apparently submissive behavior in the cricket may involve NO [178]–[183]. The cockroach was utilized by researchers in group 12 (Periplaneta Americana) and Ott et al. demonstrate that in both the cockroach and locust sGC activity can be increased via an allosteric, NO-independent mechanism as observed in mammals. An estradiol found in many animals can apparently modify NO production in the giant cockroach (Blaberus craniifer). Nitric oxide-cGMP-PKG can affect nicotinic currents in these animals and NO is implicated in MB function yet again. In fact disruption of NO production appears to impair long term but not short term memory. The modulatory Dorsal Unpaired Median cells (DUM) display increased calcium entry with increasing cGMP levels [148],[184]–[188].

## 8. Molluscan development (Groups 13–15)

Group 13 used Lymnaea stagnalis (pond snail) to study the effects of NO on development. A role was found for NO in neurite growth and synaptic re-modelling after injury but this may not be entirely due to NO acting via cGMP and PKG. Nitric oxide regulates embryonic development and affects locomotion, heartbeat and feeding. Researchers found that in the buccal ganglion NO may act both synaptically and non-synaptically in neuronal communication [189]–[191]. Group 14 chose Helisoma trivolvis (pond snail), another aquatic snail, for their research. Rehder in particular investigated growth cone function extensively using this system. Nitric oxide significantly affects growth cone filopodial pathfinding and appears to act as a chemotactic agent itself. This response involves the standard sGC-cGMP-PKG pathway including an increase in intracellular calcium from intracellular sources. Additionally NO acting as a 3-D volume transmitter affects neurons via apamin-sensitive potassium channels. Nitric oxide also produced ADP-ribosylation and affected ciliary function in the embryo [122],[192]–[201]. Finally group 15 used the marine mollusc Ilyanassa obsolete; Gifondorwa and Leise demonstrate NO is involved in both metamorphosis and apoptosis in this animal [202],[203].

## 9. Molluscan physiology (Groups 16–20)

The next 5 groups summarize the direct effects of NO on neurons and nervous systems of selected molluscs. In this section there are sufficient numbers of significant publications to separate the species even more than for development; accordingly group 16 only covers land snails while pond and marine snails are covered subsequently in separate groups (Helix pomatia, Cepaea nemoralis, Helix lucorum, Helix aspersa, Megalobulimus abbreviates). The range of NO related research is quite extensive with publications ranging from NO interacting with peptidergic transmission (FMRFamide and GSPYFVamide) to the relationship between iron metabolism and NO to the effect of magnetic fields on opioid analgesia in which NO is implicated. Again NO is implicated in the response to hypoxia, cold and nociception. It affects the type of response to glutamate on N-methyl-D-aspartate (NMDA) receptors. Additionally NO is implicated as a secondary messenger for serotonin or even a co-transmitter and may act as a retrograde transmitter as well. Nitric oxide-generated cGMP can modulate the effect of an eicosanoid on cholinergic receptor function; it decreases an inward depolarizing current. Behaviorally NO is implicated in olfaction, memory formation, rest versus activity and the withdrawal reflex. Both PKA and PKG are probably involved in olfactory behavior in the procerebrum. Finally, as mentioned previously, the author performed his PhD thesis on Helix aspersa and this was aided immensely by the extensive mapping paper published by Kerkut et al. in the mid-seventies [204]. Unlike mammalian nervous systems, the simplicity and peripheral arrangement of large bodied neurons in molluscan nervous systems facilitates the production of such a resource and apart from being able to identify a particular neuron by its position and size, one now knew the pharmacological profile of that neuron which could confirm identity. The author used F1 which was a large, easily identifiable neuron to firstly confirm that NO could be produced endogenously and then, using NO-donors, to observe any direct effect on membrane potential. Diaphorase histochemistry and immunocytochemistry had previously demonstrated the presence of NOS in the nervous system of Helix aspersa [205],[206]. The author confirmed the ability of neurons near to F1, and possibly F1 itself, to produce NO caused by acetylcholine-induced depolarization resulting in calcium entry and subsequent stimulation of NOS using the fluorescent NO reporter 4-amino-5-methylamino-2′,7′-difluorofluorescein (DAF-FM). The author then went on to show NO and membrane-permeable cGMP appeared to have a direct hyperpolarizing effect on F1 and might interact with dopamine-induced hyperpolarization. This is interesting when one compares to results obtained from Helix pomatia where the NO-cGMP system appears to decrease a calcium-activated potassium current so increasing excitability. As in many insect papers previously discussed, the 3-D volume effect was cited as particularly important for NO's functions in these animals' nervous systems [207]–[227]. Group 17 used the land gastropod Limax (L. maximus, L. valentianus, L. marginatus) to investigate the effects of NO on neural tissue. In particular these animals have been used to investigate odor processing in the procerebrum with researchers such as Watanabe and Gelperin demonstrating the importance of NO in this function. The NO-cGMP system is thought to be involved in synchronizing system oscillations necessary for discrimination and learning and memory [228]–[235]. Group 18 focused on the pond snails Lymnaea stagnalis, Helisoma trivolvis and Planorbarius corneus as model animals. Feeding behavior was studied in this group and NO-cGMP are strongly implicated as they are in certain types of learning and memory and conditioning. Additionally the buccal ganglion, which regulates gut motility, is modulated by NO; rhythmic activity of these neurons is affected. Again NO is implicated in the response to hypoxia and linked to iron metabolism. Nitric oxide appears to modulate serotinergic synaptic transmission and may be a co-transmitter; again researchers linking effects specifically to NO's ability to act in a 3-D manner due to being a hydrophobic gas. Nitric oxide can also affect the nature of the neuronal response to glutamate and the NO-sGC-PKG pathway and ADP ribosylation can release calcium from internal stores. Nitric oxide was implicated in the regulation of neuronal excitability and the ability to fire action potentials. Finally and quite significantly, the invertebrate equivalent of microglial cells respond to bacterial lipopolysaccharides by expressing iNOS similarly to mammals [215],[226],[236]–[258]. The next group (19) used the predatory sea snail Stramonita haemastoma and looked at the ability of the CNS to produce NO via NOS expression. Expression was particularly associated with sensory afferents plus it was discovered NO is probably involved in the response to environmental stress [259],[260]. Group 20 includes the bivalve molluscs Crenomytilus grayanus, Mytilus edulis and Pecten irradians. As it might be expected with such filter feeders, they have been utilized for studies on neuroprotection from pollution, temperature stress and hypoxia in which NO has been implicated. Most interestingly though may be the utilization of Mytilus for transcutaneous electrical nerve stimulation (TENS) research; similarly to the system found in mammals, TENS causes NO production via opiate signaling which is related to a subject currently causing great social concern. In addition NO appears to be involved in regulating gill filament ciliary activity; dopaminergic inhibition is antagonized by endogenous opioids acting via novel receptors and whose effect is mimicked by NO donors [261]–[266].

## 10. Aplysia and other sea slugs (Group 21)

Although molluscs, the sea slugs/hares deserve their own group as becomes their principal genus Aplysia as the invertebrate model used extensively for research into conditioned reflexes and learning and memory by such luminaries as Eric Kandel. Pleurobranchaea californica and Onchidium are also included in this group. Applying a filter to this group to just consider significant NO publications still reveals a wide range of important topics. Again NO appears involved in some forms of feeding and this can be localized to some extent to the buccal ganglion. Specifically NO is involved in swallowing and may even function in memory formation concerning swallowing. Nitric oxide is also implicated in preparing for egg laying. Nitric oxide possibly produced by interneurons can affect synaptic transmission, especially between sensory neurons and motorneurons; chemosensory areas of the mouth show NOS expression. Additionally it appears histamine and NO are likely co-transmitters. Sung et al. looked at the phenomenon of long-term hyperexcitability induced by axotomy which involves the NO-sGC-PKG pathway. This could potentially be a model for studying neuropathic pain targets. This group in particular is also a treasure trove of direct effects of NO on neurons. Nitric oxide apparently can inhibit or enhance inward depolarizing Na^+^ currents and potentiates a cAMP-mediated cation current. Nitric oxide also affects acetylcholine-(K^+^), dopamine-(K^+^), met-encephalin-(K^+^) and glutamate-induced (Cl^−^) hyperpolarizing currents principally via cGMP. It was found that NO can also modulate acetylcholine release at synapses [267]–[289].

## 11. Crustacean development (Group 22)

This group involves development studies using the lobster Homarus americanus. In this animal NO is involved in development, especially of the olfactory system and in its response to injury. At hatching expression of NOS and sGC was particularly noticeable in the olfactory system and many neurons responded to NO by increasing cGMP production suggesting the standard NO-cGMP pathway. Interestingly neurons in the stomatogastric ganglion become responsive to NO at metamorphosis during which the nervous system is completely re-organized. Elevated cGMP was also observed in the cell nuclei possibly suggesting a role in transcription [290],[291].

## 12. Crustacean physiology (Groups 23 to 26)

These final groups bring together research on the physiological effects of NO on various selected crustaceans including the lobster Homarus americanus, Pacifastacus leniusculus (crayfish), Calanus finmarchicus (zooplankton) and various crabs including Cancer productus, Neohelice granulate, Hemigrapsus sanguineus and Cancer pagurus. In the lobster (group 23) NO appears involved in feedback in neuropeptide control of the heart whereby NO released by heart tissue affects neurons in the cardiac ganglion possibly in a retrograde manner [292]–[295]. In various crabs (group 24) pigment dispersal in the retina appears to depend on NO. Nitric oxide is also implicated in processing nociceptive stimuli and the so-called gastric mill in the stomatogastric ganglion's spontaneous activity is significantly affected by NO. It should be noted that a membrane-located GC was found in this ganglion [296]–[301]. In crayfish (group 25) NO appears to be involved in glial cell apoptosis induced by photodynamic therapy (PDT) which is used in cancer treatment. Again NO is implicated in sensory perception, especially if plasticity is involved. Additionally NO and cAMP are involved in regulating swimmeret motorneurons and NO is implicated as a retrograde transmitter at NMJ's where it appears to act presynaptically. For all these actions the standard NO-sCG-cGMP pathway appears to be the principal mechanism [302]–[313]. Finally there has even been some published research to suggest functions of the various gaseous transmitters (NO, CO and H_2_S) in zooplankton (group 26) [314]. After this brief survey the final section of this review will attempt to discuss some of the more important common functions of NO in these wonderful and intriguing animals.

## 13. What these wonderful animals have taught us about nitric oxide

Some of the obvious advantages to using invertebrate models such as those listed here have already been mentioned. Accessibility, size, relative simplicity and economy are all worth considering when compared to mammalian models especially when many basic neuronal phenomena appear to have common or similar mechanisms. For the vast majority of NO's actions the same basic pathway of NO acting on sGC to produce the secondary messenger cGMP and acting on targets typified by PKG appears to be the common mechanism. However ADP-ribosylation should not be ignored as a potential means to affect the function of many proteins. Additionally, in many of these organisms NO has been strongly implicated as a retrograde synaptic transmitter. For development affected by NO in insects (groups 1–4) these model organisms have enabled us to video and directly observe neuronal development, including that of the behaviorally significant central complex. Nitric oxide is implicated in embryogenesis, neurogenesis and synaptogenesis. Development in molluscs (groups 13–15) appears similarly affected by NO. Again embryogenesis and metamorphosis are affected together with cellular-level events such as neurite growth and apoptosis. Here, as in many areas, the fact that NO is a gas and can act 3-D volume transmitter appears crucial to its function. As for development in crustaceans (group 22), lobsters also need NO for metamorphosis and development of the olfactory system. Summarizing NO's physiological actions on neurons, in insects (groups 5–12) it is strongly implicated in associative learning and in various stages of memory formation as well as regulating heart function. Nitric oxide is also implicated in sensory function, in particular olfactory discrimination; again NO acting as a 3-D volume transmitter appears crucial to its function. Molluscan neurophysiology (groups 16–21) has demonstrated that NO is implicated in neuropeptide function, iron metabolism, responses to hypoxia, cold and nociception. Again it is implicated in olfaction and odor processing and some types of learning and memory. Finally in crustaceans (groups 23–26) NO can affect the heart and gut and is implicated in sensory perception and apoptosis. Hopefully this brief summary has helped illustrate how useful invertebrate model systems can be when examining the function of such a novel messenger molecule as NO.
